# Neofunctionalization in Vertebrates: The Example of Retinoic Acid Receptors

**DOI:** 10.1371/journal.pgen.0020102

**Published:** 2006-07-14

**Authors:** Hector Escriva, Stéphanie Bertrand, Pierre Germain, Marc Robinson-Rechavi, Muriel Umbhauer, Jérôme Cartry, Marilyne Duffraisse, Linda Holland, Hinrich Gronemeyer, Vincent Laudet

**Affiliations:** 1 Structure and Evolution of Nuclear Hormone Receptors, UMR 5161 du CNRS, INRA LA 1237, Laboratoire de Biologie Moléculaire de la Cellule, IFR128 BioSciences Lyon-Gerland, Ecole Normale Supérieure de Lyon, Lyon, France; 2 Institut de Génétique et de Biologie Moléculaire et Cellulaire (IGBMC), CNRS/INSERM/ULP/Collège de France, BP 163, Illkrich, CU de Strasbourg, France; 3 UMR CNRS 7622, Biologie du Développement, Case 24, Université Pierre et Marie Curie, Paris, France; 4 Marine Biology Research Division, Scripps Institution of Oceanography, University of California San Diego, La Jolla, California, United States of America; Princeton University, United States of America

## Abstract

Understanding the role of gene duplications in establishing vertebrate innovations is one of the main challenges of Evo-Devo (evolution of development) studies. Data on evolutionary changes in gene expression (i.e., evolution of transcription factor-*cis*-regulatory elements relationships) tell only part of the story; protein function, best studied by biochemical and functional assays, can also change. In this study, we have investigated how gene duplication has affected both the expression and the ligand-binding specificity of retinoic acid receptors (RARs), which play a major role in chordate embryonic development. Mammals have three paralogous *RAR* genes—*RARα, β,* and *γ*—which resulted from genome duplications at the origin of vertebrates. By using pharmacological ligands selective for specific paralogues, we have studied the ligand-binding capacities of RARs from diverse chordates species. We have found that RARβ-like binding selectivity is a synapomorphy of all chordate RARs, including a reconstructed synthetic RAR representing the receptor present in the ancestor of chordates. Moreover, comparison of expression patterns of the cephalochordate amphioxus and the vertebrates suggests that, of all the RARs, RARβ expression has remained most similar to that of the ancestral RAR. On the basis of these results together, we suggest that while RARβ kept the ancestral RAR role, RARα and RARγ diverged both in ligand-binding capacity and in expression patterns. We thus suggest that neofunctionalization occurred at both the expression and the functional levels to shape RAR roles during development in vertebrates.

## Synopsis

In eukaryotic organisms, each gene is a stretch of DNA composed of control regions that bind transcription factors and coding regions that transcribe the mRNA that is later translated into proteins. At the molecular level, changes in control regions can affect the time and place at which a protein is synthesized, whereas changes in the coding region can alter the protein's function. Retinoic acid receptors (RARs) are chordate-specific transcription factors which, upon binding the natural morphogen retinoic acid, bind to and activate transcription from target genes. Here, the authors show how the ligand specificity of RARs has changed during vertebrate evolution in parallel with changes in expression. Through functional characterization of the RARs from several vertebrates, the chordate amphioxus, and the reconstructed ancestral RAR sequence, the authors show that of the three vertebrate RARs, RARβ has retained the ancestral characteristics in terms of both function and expression, while RARα and γ have evolved by acquiring new functions, both new binding specificity and new expression patterns. Thus both types of evolution have been important in the diversification of vertebrate RARs.

## Introduction

The origin of organismal complexity is generally thought to be tightly linked to the evolution of new gene functions. Susumu Ohno proposed in 1970 that, in contrast to mutations, gene duplications can create evolutionary novelties [1]. He also proposed, based on the genome weight of different deuterostomes, that two periods of genome duplication occurred during evolution of the vertebrate lineage [1]. This hypothesis has been revisited and discussed by different authors, and even if the precise timing and mechanisms of these gene duplications are still under discussion, their general importance during vertebrate evolution is now widely accepted [2–4].

**Figure 1:**
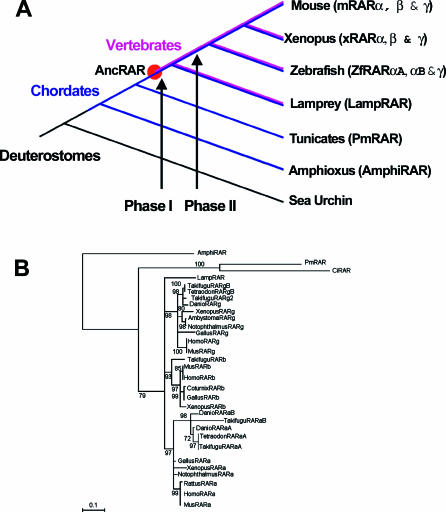
Phylogenetic View of Deuterostomes and RARs (A) Current view of deuterostome phylogeny with amphioxus representing the basal chordate [5]. RARs used in the present study are indicated at their respective taxonomic positions—for mouse, *Xenopus,* zebrafish, lamprey, amphioxus, and tunicates. The position of the synthetic ancestral sequence is indicated by a red circle. The two proposed periods of whole genome duplications in vertebrates are indicated as Phase I and Phase II, occurring respectively before and after the divergence of lampreys. (B) Phylogenetic tree showing the placement of the RARs used in this study. Branch length is proportional to evolutionary change (bar = 0.1 substitutions per site); numbers at nodes are bootstrap support, in percent of 1,000 replicates. Branches supported by bootstrap lower than 70% have been polytomised. The tree was rooted by the amphioxus sequence, in agreement with [5]. Species abbreviations and their groups are indicated as follows. Amphioxus: Amphi, *Branchiostoma floridae.* Tunicates: Pm, *Polyandrocarpa misakiensis;* Ci, *Ciona intestinalis.* Lampreys: Lamp, Petromyzon marinus. Teleost fish: Takifugu, *Takifugu rubripes;* Tetraodon, *Tetraodon nigroviridis;* and Danio, Danio rerio. Amphibians: Xenopus, *Xenopus laevis;* Ambystoma, *Ambystoma mexicanum;* and Notophthalmus, *Notophthalmus viridescens.* Birds: Gallus, *Gallus gallus;* and Coturnix, *Coturnix coturnix.* Mammals: Homo, *Homo sapiens;* Mus, *Mus musculus;* and Rattus, Rattus norvegicus.

Cephalochordates (specifically the small marine animals called amphioxus) have been generally considered the closest extant invertebrates to vertebrates. Although recent studies place urochordates as the sister group of vertebrates [5,6], it remains accepted that amphioxus diverged from the vertebrate lineage before the vertebrate genome duplications occurred (Figure 1A). In general, for each gene paralogy group in vertebrates, amphioxus contains a single copy of its respective orthologue—amphioxus contains a single *Hox*-cluster [7] instead of four in mammals, and a single retinoic acid receptor (RAR), AmphiRAR [8], instead of three as in mammals (RARα, RARβ and RARγ). Many data suggest that the duplications took place at two distinct periods during evolution, one before the split of agnathans (hagfish and lampreys) and one before the split of cartilaginous fishes [9–11]. The lamprey genome has probably experienced only one of these large-scale gene duplications, although some independent duplications also occurred in the lamprey *Hox*-cluster [12].

The contribution of duplicated genes to the origin of evolutionary novelties has been formalized by the “duplication-degeneration-complementation” model [13]. This model establishes three possible fates for duplicate genes: (i) one member of the duplicated pair degenerates by accumulating deleterious mutations, while the other retains the original gene function; (ii) the ancestral function is partitioned and shared by the two members of the duplicated pair (subfunctionalization); or (iii) one duplicate acquires a new function while the other retains the original function (neofunctionalization).

Two paths for the generation of evolutionary novelties have been proposed: (i) changes in the noncoding moiety of the gene (i.e., evolution of *cis*-regulatory elements) and (ii) changes in the coding moiety of the gene (i.e., evolution of protein function). Changes in transcriptional regulation of the genes can underlie the evolution of body plan diversity. Thus, spatial and temporal changes in gene expression of orthologous *Hox* genes in different vertebrates are correlated with morphological innovations. For example, a change in the expression domains of *Hox* genes correlates with anatomical differences among vertebrae in tetrapods [14]. Similarly, the three mammalian RARs have overlapping but somewhat different expression domains and their functions are not entirely redundant [15,16]. Sequence changes in proteins and consequent alterations in their biochemical functions could also underlie the diversification of body patterns. For example, changes in DNA-binding specificity of a transcription factor, its interactions with cofactors, or the posttranslational regulation of its activity could evolve in concert with more complex developmental roles (reviewed in [17]). Unfortunately, given the experimental limitations in characterizing the protein functions of developmental genes, little evidence to date supports the functional diversification of relevant genes during the chordate-to-vertebrate transition. RARs are particularly well suited for such a goal, since their developmental function is well studied and it is possible to characterize their DNA- and ligand-binding properties as well as their transcriptional and dimerisation activities [18]. Thus, RARs combine the potential for both classical Evo-Devo (evolution of development) and “Evo-Fun” (evolution of function) studies.

**Figure 2:**
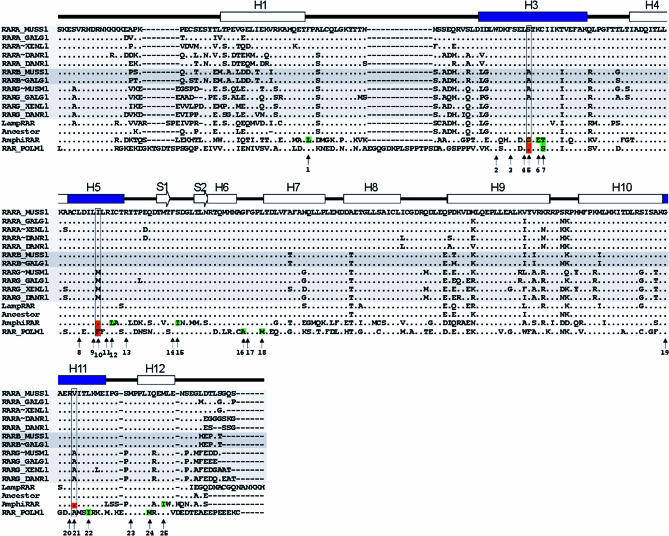
Protein Sequence Alignment of Selected Gnathostome RARs RARs are represented from lamprey (LampRAR, Petromyzon marinus), amphioxus (AmphiRAR, Branchiostoma floridae), tunicate (RAR_POLM1, Polyandrocarpa misakiensis), and the synthetic predicted ancestral RAR (Ancestor). The position of the 12 helices is indicated above the alignment (H1–H12). Residues implicated in direct contacts with the ligand are numbered from 1 to 25 below the alignment. The three divergent residues within the LBP between vertebrate RARs are within vertical rectangles in helices 3, 5, and 11. Gnathostome and *Polyandrocarpa* sequences are named with the nomenclature code used in the nuclear receptor database NUREBASE (http://www.ens-lyon.fr/LBMC/laudet/nurebase/nurebase.html) [39].

To decipher how gene duplications affected the ligand binding function, in the present work we studied RARs of several chordates as well as a reconstructed RAR representing the hypothetical sequence present in the ancestor of all vertebrates (AncRAR, Figure 1A; Table 1). The ligand-binding domain (LBD) of nuclear receptors, including RARs, is structured in a three-layered α-helical antiparallel sandwich of 12 helices (H1–H12), forming a hydrophobic ligand-binding pocket (LBP, Figure 2). In RARs, this LBD is composed of about 270 amino-acids, with about 25 localized in H1, H3, H5, the β-turn, loop 6–7, H11, loop 11–12, and H12, which all make direct contact with the ligand (Figure 2). To date, only one in vivo ligand of all vertebrate RARs is known—all-*trans* retinoic acid (ATRA)—and genetic evidence in mice has suggested that retinoic acid (RA) metabolites do not play a significant developmental role [19]. However, the LBPs of human RARs differ from each other in three amino acid positions [20,21], which cause different binding specificities in vitro, with differential binding and transactivation of each paralogue induced by different synthetic retinoids [22]. It is not known whether this difference in specificity has a role in vivo. Since the ligand-binding selectivity and the LBP structure are directly correlated [23], we used these synthetic retinoids as markers of the LBP structure in RARs. Thus, the comparison of the ligand-binding abilities of different RARs from organisms at key phylogenetic positions and of RARs with mutated LBPs provides information about the evolution of LBP structure and function.

**Table 1:**
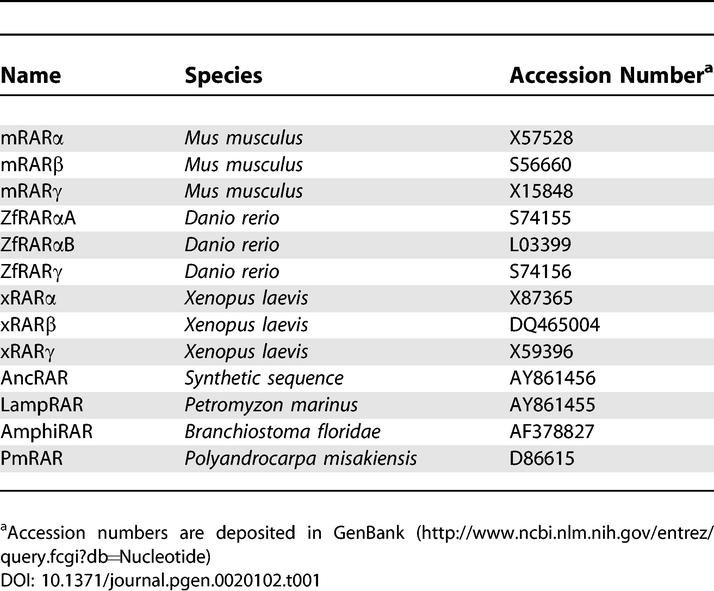
cDNA Sequences of RARs Used in the Present Study

To explore how gene function evolved in vertebrates after gene duplications we studied and compared two properties of RARs in different chordates to decipher changes in the coding and non coding moieties of the gene during evolution—namely, the ligand-binding capacity and the gene expression pattern during embryonic development, respectively.

## Results

### Vertebrate RARs Arose from Duplications at the Origin of Vertebrates

The phylogeny of RARs (Figure 1B), including xRARβ *(Xenopus),* the first report of an *RARβ* gene outside amniotes, is consistent with known chordate phylogeny, and with the hypothesis that *RARα, β,* and *γ* arose from duplications at the origin of vertebrates. Although a number of branchings are not well resolved, support for the nodes that are important to our discussion is strong (RARα, β, γ are supported by bootstrap > 90%).

### RARs Bind ATRA in All Chordates

**Figure 3:**
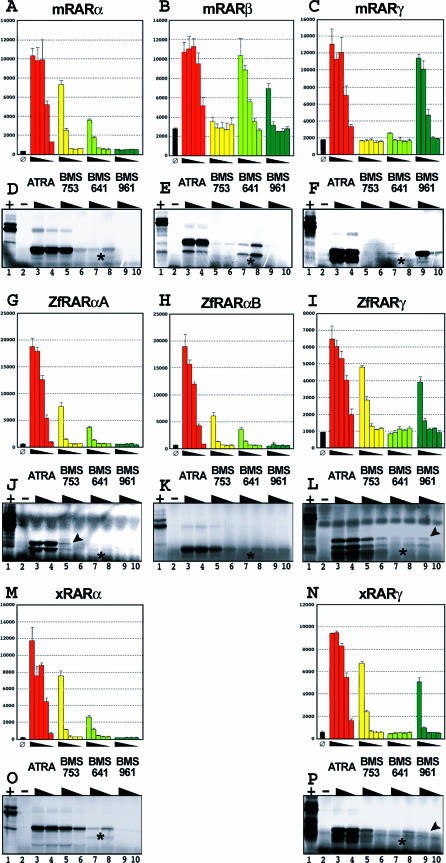
Transcriptional Activity and Binding Selectivity of Vertebrate RARs Transcriptional activity is shown in (A–C), (G–I), (M), and (N), and corresponding binding selectivity in (D–F), (J–L), (O), and (P). Identities of the vertebrate RARs for each activity-selectivity pair are indicated above each bar graph. In each case, a chimera comprising the RAR LBD fused to the GAL4 DNA-binding domain (GAL-RAR(LBD)) has been used. The analysis of transcriptional activity in (A–C), (G–I), (M), and (N) shows transient transactivation assays in Cos1 cells with the indicated GAL-RAR(LBD) expression vector and the cognate (17m)5x-G-luc reporter plasmid, in the presence of increasing concentrations (10^−10^ to 10^−6^ M) of ATRA (red bars), BMS753 (yellow bars), BMS641 (light green bars), and BMS961 (dark green bars) respectively. The black bars indicate transactivation in the absence of hormone. Partial proteolysis maps of different in vitro-translated RARs are shown in (D–F), (J–L), (O), and (P). For each proteolysis gel lane 1 represents the undigested protein, lane 2 shows digestion of the receptor in the absence of ligand, lanes 3 and 4 show digestion of the receptor in the presence of ATRA (10^−4^ to 10^−5^ M), lanes 5 and 6 show digestion in the presence of BMS753 (10^−4^ to 10^−5^ M), lanes 7 and 8 show digestion in the presence of BMS641 (10^−4^ to 10^−5^ M), and lanes 9 and 10 show digestion in the presence of BMS961 (10^−4^ to 10^−5^ M). Protected bands in the presence of BMS641 are indicated by an asterisk, and slightly protected bands are indicated by arrowheads.

**Figure 4:**
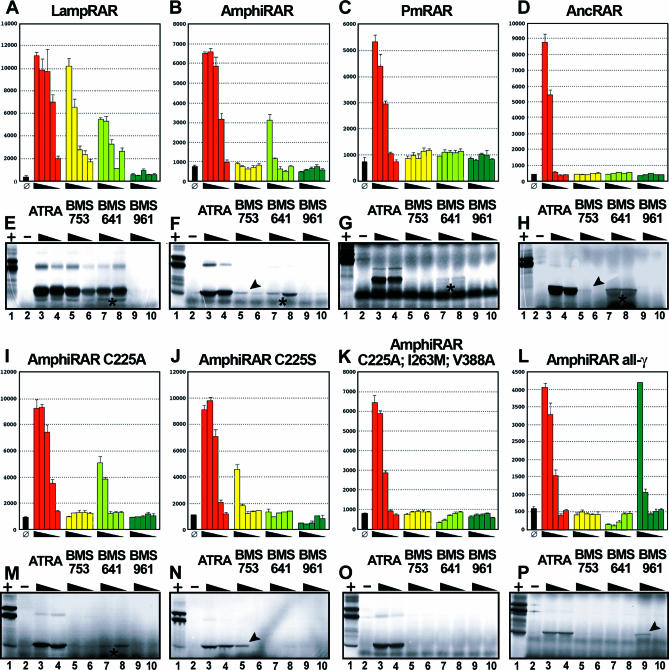
Transcriptional Activity and Binding Selectivity of Chordate RARs Transcriptional activity is shown in (A–D) and (I–L), and corresponding binding selectivity in (E–H) and (M–P). Identities of the chordate RARs for each activity-selectivity pair are indicated above each bar graph. Transcriptional activity is shown in (A–D) for LampRAR, AmphiRAR, PmRAR, and AncRAR, and that of AmphiRAR mutants is shown in (I–L). Partial proteolysis maps of the different in vitro-translated RARs are shown in (E–H) and (M–P). Chimeric GAL-RAR(LBD) transactivation methods, colour code of the transactivation figures, and contents of each proteolysis gel are as in Figure 3. Protected bands in the presence of BMS641 are indicated by an asterisk, and slightly protected bands are indicated by arrowheads.

Transactivation of a luciferase reporter gene with Gal4-RAR(LBD) constructs in transient transfections, in parallel with a limited proteolysis assay, was used to ascertain both transcriptional and ligand-binding activities of the receptors. Our results show that all the chordate RARs, including the reconstructed AncRAR, are able to transactivate in a dose-dependent manner with ATRA (red bars, Figures 3 and 4). The EC_50_ values (~10^−9^ to 10^−8^ M) for all the chordate RARs are in a similar range, suggesting that binding of ATRA to RAR is a shared ancestral function in all chordates.

### The Structure of the LBP Directs the Differential Recognition of Synthetic Monospecific Retinoids

As previously shown, only three amino acid positions differ between the LBPs of mammalian RARs [22]. These positions account for the different binding specificities with synthetic retinoids (various Bristol-Myers Squibb synthesized retinoids [BMS] compounds [22,24]) to the receptors (Figures 3 and S1A). However, the LBP of each RAR differs between several vertebrate orthologues. For example, RARγs of zebrafish and *Xenopus* have an amino acid in H3 that is found in mammalian RARα (Ser) but not in mRARγ (mouse; Ala), while the two other positions are the same as those of mRARγ (Met and Ala) (see Figures 2 and S1A). Since the overall structure of the receptor can influence the LBP, we tested the selective ligand recognition of zebrafish and *Xenopus* RARs (Table 1). Transactivation assays in the presence of increasing concentrations of the BMS compounds showed that all the RARαs from vertebrates that share the same three key amino acid positions (Ser, Ile, Val) have a comparable transactivation pattern as the synthetic compounds (Figure 3A, 3G, 3H, and 3M) (i.e., high transactivation with BMS753 and low transactivation with BMS641). However, xRARγ and zfRARγ (zebrafish), which differ at a key amino acid position (Ser in H3) from mRARγ (Ala in H3) (see Figure S1A), exhibit a pattern intermediate between that of mRARα and mRARγ (Figure 3I, 3L, 3N, and 3P)—they transactivate with both BMS753 and BMS961. We noted that the binding abilities of the receptors tested by a limited proteolytic digestion always paralleled the transactivation patterns except for the BMS641 compound (Figure 3D–3F, 3J–3L, 3O, and 3P), since all the vertebrate RARs can bind this retinoid, but only RARβ, and to a lesser extent RARα, can transactivate in its presence.

These data suggest that changes in the LBPs of RARs may have played a functional role during vertebrate evolution. They also show that the use of transactivation and/or binding assays in the presence of synthetic monospecific compounds is an excellent tool for studying the structure-function relationships of different RARs, and potentially of other nuclear receptors, since the transactivation and binding pattern reflect the LBP structure.

### Chordate RARs Share an LBD Structure Able to Bind At Least ATRA and the β-Specific Compound BMS641

AmphiRAR diverged evolutionarily before the vertebrate-specific genome duplications occurred and represents one of the closest invertebrate RARs to the vertebrate RARs (Figure 1). The AmphiRAR sequence has a high percentage of identity with the vertebrate RARs (~88% DNA-binding domain, ~58% LBD). As previously shown, AmphiRAR functions in a dose-response manner in the presence of ATRA (Figure 4) [8]. However, one of the three key amino acid positions within the LBP of AmphiRAR (C225, I263, and V388, Figure S1A) diverges from those of vertebrate RARs (Figures 2 and S1A): the position at H3 (Cys) does not correspond to any of the three vertebrate RARs, while the two other key positions (H5 and H11) are conserved with both the mammalian α and β paralogues (Ile and Val). With the synthetic monospecific retinoids, AmphiRAR is able to transactivate the reporter gene in the presence of the mammalian β-specific compound (BMS641) (Figure 4B), which it binds strongly. It also binds the α-specific compound (BMS753) (Figure 4F). This is reminiscent of the specificity exhibited by mRARβ, suggesting that AmphiRAR and mRARβ LBPs share a similar structure (compare Figure 3B and 3E with Figure 4B and 4F).

Although both the tunicate RAR (PmRAR) and the ancestral RAR (AncRAR) transactivate the reporter gene in the presence of increasing amounts of ATRA, neither is able to activate transcription in mammalian cells in the presence of any synthetic monospecific retinoid (Figure 4C and 4D). However, both PmRAR and AncRAR are able to bind weakly the β-specific retinoid (BMS641) (asterisks, Figure 4G and 4H), suggesting once again that a similar structure of the LBD is shared by mRARβ, PmRAR, and AncRAR (compare Figure 3E with Figure 4G and 4H).

LampRAR is able to bind and transactivate the reporter gene in the presence of increasing concentrations of ATRA and the synthetic compounds BMS753 and BMS641 (Figure 4A and 4E). Twenty-five residues within the LBP of RARs, including the three variable residues of RARα, β, and γ in mammals, make direct contact with the ligand [20] (Figures 2 and S2). These 25 positions are strictly conserved between LampRAR LBP and mRARα. However, the LampRAR transactivation and binding pattern in the presence of the BMS compounds is a composite of those of mRARα and mRARβ (i.e., high transactivation and binding with both BMS753 and BMS641; compare Figure 3A and 3D with Figure 4A and 4E). This result suggests that the overall structure of the receptor can influence the LBP.

### Vertebrate RARs Acquired Different Monospecific Ligand Specificities by Accumulating Mutations in Their LBPs following Gene Duplications

It is known that single point mutations at the three key positions of the LBP of mammalian RARs suffice to change their specificities for the synthetic monospecific retinoids [22]. Since only the first of the three key positions of the AmphiRAR (Cys225) is divergent compared to mRARα and β, we mutated it either to Ser (like the corresponding position in mRARα) or to Ala (like the corresponding position in mRARβ) and determined the capacity of the mutant proteins to bind different synthetic monospecific retinoids. We also asked whether mutating Cys-Ile-Val to Ala-Met-Ala (like the corresponding positions in mRARγ) would confer a γ-like specificity to AmphiRAR. The two first mutants (C225A and C225S) conferred α-like and β-like transactivation and binding patterns respectively, (Figure 4I, 4J, 4M, and 4N). However, the triple mutant (C225A, I263M, V388A) did not confer the γ-like pattern. Instead, this mutant completely lost its capacity to bind any of the monospecific retinoids (Figure 4K and 4O). When we compared the sequence of the 25 amino acid positions of the LBP in AmphiRAR and mRARγ LBPs, we found that the AmphiRAR LBP contains not three but nine divergent positions (seven when compared to mRARα and mRARβ; see Figures 2 and S2). Mutating all of these nine positions to those of the mRARγ LBP recovered the BMS γ-like binding behaviour (Figure 4L and 4P). These results show that a relatively small number of mutations in key residues of the LBP can change the specificity of the RARs.

### Vertebrate RARs Show New Expression Territories When Compared with AmphiRAR

**Figure 5:**
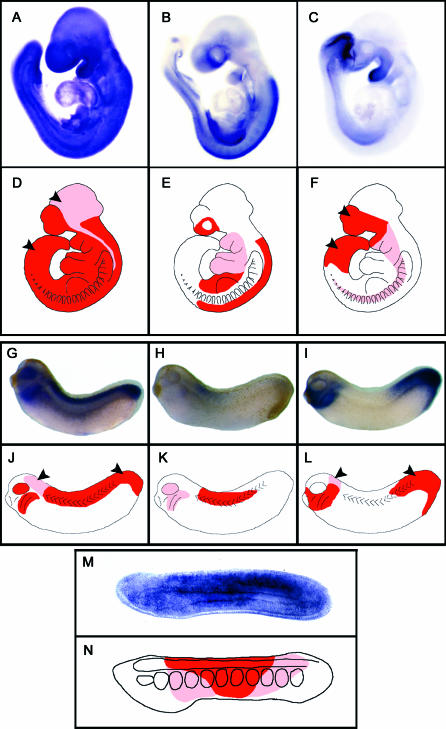
Schematic Representation of the Expression Territories of RARs Staining of embryos indicates expression of mRARα (A), mRARβ (B), and mRARγ (C) in mouse embryos at E9; of xRARα (G), xRARβ (H), and xRARγ (I) in stage 30 *Xenopus* embryos, and of AmphiRAR (M) in 20 h old amphioxus larvae. Schematic representations are shown of the expression territories of mRARs (D–F), xRARs (J–L), and AmphiRAR (N) in mouse, *Xenopus,* and amphioxus embryos, respectively. Regions with high levels of expression are red and those with lower levels of expression are pink. Arrowheads indicate regions in mouse and *Xenopus* embryos where the RAR expression cannot be correlated with AmphiRAR expression and can be described as “new expression territories.”

We previously showed that during amphioxus development, AmphiRAR is strongly expressed at 16 to 24 hours postfertilization in the middle third of the neural tube, somites, and endoderm but not in the cerebral vesicle or notochord [8]. Thus AmphiRAR gene expression decreases strongly at the anterior and posterior parts of the larvae [8] (Figures 5 and S3). Expression of the three mammalian and *Xenopus* RARs at comparable stages (at embryonic day 9 [E9] in mouse and at stage 30 in *Xenopus*) is diagrammed in Figure 5. In mouse, RARα is ubiquitously expressed but is at particularly high levels in both the neuroectoderm and mesenchyme of the head, RARγ is strongly expressed in the tail and forebrain, and RARβ transcripts are present in the head mesenchyme, in the trunk tissues, and in the mesonephric duct, but are not detectable in the forebrain and tail (Figures 5A–5F and S4) [15,16]. A comparable gene expression pattern is observed in *Xenopus,* with a ubiquitous expression of RARα (especially in the neuroectoderm and head regions), a polarized expression of RARγ in the brain and posterior parts of the embryo, and expression of RARβ in the posterior hindbrain and anterior spinal chord, as well as in branchial arches (Figures 5G–5L and S5).

## Discussion

**Figure 6:**
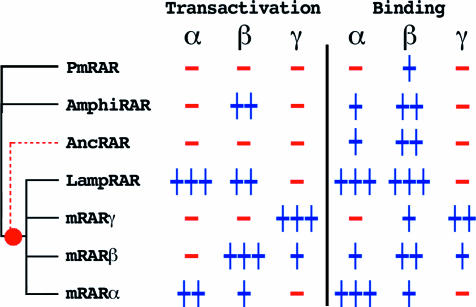
Representation of the Transactivation and Binding Capacities of the RARs Used in the Present Study The three synthetic retinoids are shown as α, BMS753; β, BMS641; and γ, BMS961. The phylogenetic relationships between the RARs have been schematized by a phylogenetic tree (the tunicate and amphioxus RARs have been polytomised, LampRAR is also polytomised with the vertebrate RARs). The putative position in the tree of the ancestral sequence is indicated by a dashed branch in red.

A general overview of the binding and transactivation capacities of chordate RARs is shown in Figure 6. Despite their different transactivation patterns, all the chordate RARs bind the β-specific retinoid BMS641. This suggests that the LBPs of all the chordate RARs share common features. Using AmphiRAR as a model, we have also shown that just a few mutations in the LBP are sufficient to change the binding selectivity of the receptor. Even if possible evolutionary scenarios can be drawn in which a position mutates back and forth between two alternative amino acids, the demonstration of the presence of endogenous RA in amphioxus, the high-affinity binding of ATRA to AmphiRAR, and the activity of ATRA during amphioxus embryonic development [8,25] lead us to propose that the most parsimonious explanation of all these results is that chordate RARs evolved from a common ancestor that was already able to bind ATRA and had an LBP similar to that of modern mammalian RARβ. This model has two interesting implications. First, the mammalian *RARβ* gene has conserved structural and functional aspects of the ancestral *RAR*. Second, following the vertebrate-specific genome duplications, other vertebrate *RAR* genes accumulated mutations in the LBP that changed the structure and the specificity of the protein they encode. Of note, each vertebrate lineage evolved differently, since fish and *Xenopus* RARs have different structure-function relationships from those of the mammalian RARs. Thus, of the three paralogues resulting from vertebrate genome duplications, RARβ retained the ancestral binding specificity, leaving RARα and γ free to “explore” other functionalities. An important constraint on RAR evolution was that all paralogues had to bind the major ligand ATRA. In addition to this constraint, it appears that one duplicate, RARβ, kept the ancestral LBP functionality. This allowed the other paralogues to be selected for new functions. While RARα remained relatively close to the ancestral RAR (weak transactivation by BMS641), RARγ evolved the most divergent LBP (nine point mutations needed to recover a γ-like functional LBP in AmphiRAR [Figure 4L and 4P]). Phylogenetic analysis confirms unambiguously that the three paralogues RARα, β, and γ result from vertebrate-specific gene duplications after the divergence from tunicates and amphioxus and before that of teleost fish (Figure 1B). Resolution of the order of duplication events, and the exact position of LampRAR, is less clear, since support for the different topologies of the tree is limited (< 70% bootstrap), perhaps because of rapid evolution of RARγ and of the outgroup RARs.

### Is the RAR Ligand-Binding Specificity the Only Functional Characteristic That Changed following the Vertebrate-Specific Gene Duplications?

Just as mRARβ and AmphiRAR have similar ligand-binding selectivity, they also have similar expression patterns. In contrast, although mRARα and γ share some expression domains with RARβ, they are also expressed in other embryonic territories (forebrain and caudal regions). Thus, expression of amphioxus RAR is either repressed in the anterior and posterior parts of the embryo and this repression has been lost in vertebrate RARα and γ, or activation of these receptors in anterior and posterior tissues has been acquired during vertebrate evolution.

Moreover, we have shown that RA directly patterns the pharyngeal endoderm in amphioxus, an invertebrate that lacks neural crest [8,26]. In mammals a role of RA during development of the branchial region was long ascribed to defects in the migration of the neural crest cells. However, it has been demonstrated with isoform-specific retinoids and knockout mice that RA has a direct role on development of the branchial region in the mouse, and that this function is carried exclusively by RARβ and not RARα or γ [27]. This shows that RARβ has not only conserved the expression pattern and the ligand-binding selectivity of the ancestral RAR but also a central biological role during embryonic development.

Taken together, these findings support a model in which an ancestral RAR containing an LBP close to that of mRARβ was expressed in the trunk region of the putative ancestral chordate. This ancestral RAR patterned the anterior-posterior axis of both the neuroectoderm and endoderm. Following vertebrate-specific gene duplications, neofunctionalization events generated new RAR functions. One vertebrate paralogue, RARβ, was constrained by natural selection and kept most of the ancestral functions, allowing the two other paralogues to take on new possible functions. Thus, RARα and γ gained new expression territories (forebrain and tail regions of the embryo) and, in parallel, they also diverged in their LBP structure.

Although it has been shown that oxidative derivatives of RA (i.e., degradation products) are not in vivo ligands of RARs [19], the evolutionary scenario presented here leaves open the possibility that vertebrate RARs could bind different ligands in vivo since their LBPs also evolved by neofunctionalization. Research of possible natural ligands with different affinities for each vertebrate RAR paralogue should address this question in the future.

## Materials and Methods

### Phylogenetic analysis and ancestral sequence estimation.

The RAR LBDs used are presented in Table 1. Amino acid sequences of chordate RARs were aligned using the CLUSTAL W program [28] and manually corrected with SEAVIEW [29]. Phylogenetic trees were inferred by maximum likelihood as implemented in PhyML [30] with the JTT+γ model. The 245 complete sites (no gap, no X) were used. Robustness was assessed by bootstrap analysis (1,000 repetitions) [31].

The ancestral RAR of vertebrates, before duplication, was reconstructed by maximum likelihood as implemented in PAML [32], under the JTT+γ substitution model. Sites with indels were treated as follows. (i) If the indel was due to partially sequenced genes, the partial sequences were excluded for reconstruction of these specific sites. (ii) The ancestral state (gap or not) of other indels was estimated manually by parsimony. (iii) If this parsimony analysis predicted that the sites were present in the ancestor, they were reconstructed by maximum likelihood, excluding the sequences with the deletion. Few sites in the LBD were affected by this problem. Overall, the reconstruction was very good, with an average confidence in predicted sites of 0.990.

### Cloning of LampRAR, xRARβ, and mutation constructs.

*LampRAR* was obtained by semi-nested RT-PCR with degenerate primers based on vertebrate RARs and first-strand cDNA synthesized from total RNA of Petromyzon marinus liver, brain, and muscle. *xRARβ* was obtained by PCR using specific primers based on the incomplete genome of Xenopus tropicalis found in the ENSEMBL database (http://www.ensembl.org/Xenopus_tropicalis/index.html) and first-strand cDNA synthesized from total RNA of Xenopus laevis embryos.

The Gal4 mutants correspond to a fusion between residues 1 and 147 of Gal4 and the LBDs of the different chordate RARs. The starting position for the LBD was the Ser154 for AmphiRAR (29 amino acids before H1 of the LBD; see Figure 2) and the corresponding conserved serine residues of the other chordate RARs. Mutants were constructed by PCR-assisted site-directed mutagenesis. In this procedure mutagenesis is performed by creating an oligonucleotide primer that is complementary to the normal DNA sequence except for the mutant base, which is generally positioned near the 5′ end of the oligonucleotide to ensure adequate priming. The mutant primer is incorporated by PCR into the newly synthesized DNA. This procedure is repeated as many times as mutations are introduced, and the final DNA fragment is sequenced to confirm the presence of the mutated positions. To subclone in phase with the Gal4 protein ORF, the 5′ ends of the primers for amplifying each sequence contained restriction sites corresponding to the specific insertion site of the pG4MpolyII vector [33]. The primers with the desired point mutation were designed to overlap the corresponding region within the wild-type sequence. The chimeric and mutant constructs were sequenced to confirm their identity.

### Transactivation assays in mammalian cells.

Cos-1 (monkey kidney) cells were maintained in DMEM supplemented with 5% charcoal-treated FCS. The cells were transfected at 70% confluence in 24-well plates using 4 μl of ExGen 500 (Euromedex, Souffelweyersheim, France) with 1.0 μg of total DNA including 0.1 μg of reporter plasmid (17m)x5-tk-luc, and 10 ng of CMV-βGAL as an internal control to account for variations of transfection efficiency. The culture medium was changed 6 h after transfection and, when appropriate, ATRA or the RA agonists BMS753, BMS641, and BMS961 in ethanol were added to different final concentrations (10^−10^ to 10^−6^ M). Cells were lysed 24 h after transfection and assayed for luciferase activity.

### Limited proteolytic digestion.

These assays were done as described [34] using in vitro-translated ^35^S-labelled RARs (TNT kit; Promega, Madison, Wisconsin, United States). Briefly, after incubating at room temperature for 15 min with ligands, receptor proteins were digested at room temperature for 10 min with 25 μg/ml trypsin. The proteolytic fragments were separated on a 10% SDS polyacrylamide gel and visualized by autoradiography.

### In situ hybridization.

*AmphiRAR, mRARα, mRARβ, mRARγ, xRARα, xRARβ,* and *xRARγ* partial cDNAs cloned into the pBluescript vector (Stratagene, La Jolla, California, United States) and linearized with appropriate enzymes were used for synthesis of antisense riboprobes. For *AmphiRAR,* fixation and whole-mount in situ hybridization were done as described [35]. Two probes were combined—one synthesized to the 3′ UTR plus a 735 bp probe to the 5′ end of the cDNA. For mRARs, whole-mount in situ hybridization was done using standard methods [36]. Probes correspond to the DNA-binding domain for mRARα and the LBD region for mRARβ and mRARγ. Labelling of the probes was performed using the digoxigenin-UTP labelling kit (Roche, Basel, Switzerland). Xenopus laevis eggs were obtained from females injected with 500 IU of human chorionic gonadotropin, artificially fertilized, dejellied with 2% cysteine hydrochloride (pH 7.8), and cultured in 0.1× modified Barth's saline. Embryos were staged as described [37]. In situ hybridization was carried out as previously reported in [38].

## Supporting Information

Figure S1 Conservation/Divergence of the Sequences Used in the Present Study (A) Amino acids within the LBP corresponding to the three variable positions in H3, H5, and H11 of mammalian RARs, for the chordate RARs used in this study.
(B) Distance matrix of the sequences used in the alignment shown in Figure 2. Gnathostome and *Polyandrocarpa* sequences are named with the nomenclature code used in the nuclear receptor database NUREBASE (http://www.ens-lyon.fr/LBMC/laudet/nurebase/nurebase.html) [39].
(3.8 MB TIF)

Figure S2 Schematic Representation of the 25 Residues Present in the LBP of AmphiRAR Contacting ATRA The three key residues that differ between vertebrate RARs are indicated by a circle. The other six residues that differ between mRARγ and AmphiRAR are indicated by a square. Adapted from [20].
(220 KB TIF)

Figure S3 Expression of AmphiRAR in Amphioxus Embryos Whole mounts are shown with anterior toward the left. Blastula stage shows no expression and gastrula shows ubiquitous expression (unpublished data). In early neurula (15 h) expression is down-regulated in the cerebral vesicle (arrow), anterior endoderm and non-neural ectoderm. In 18- to 22-h neurula the expression is down-regulated in the anterior third of the nerve cord and in the pharyngeal endoderm and is up-regulated in the middle third of the embryo. In 24- to 30-h embryos the expression is strong in the nerve cord posterior to the cerebral vesicle and in a small region of endoderm, but is largely down-regulated elsewhere.
(3.1 MB TIF)

Figure S4 Expression of mRARα, mRARβ, and mRARγ in Mouse Embryos from E9 to E12.5 RARα is ubiquitously expressed from E9 to E12.5 with a very high level in the anterior brain. RARβ is expressed in the central part of the CNS from E9 to E12.5 as well as in some parts of the head mesenchyme and in other trunk tissues. RARγ is mainly expressed in the forebrain, the tail, the branchial arches, and the limb buds as they develop.
(9.0 MB TIF)

Figure S5 Expression of xRARα, xRARβ, and xRARγ in *Xenopus* embryos Whole mounts are oriented with anterior toward the right. Expression of RARα and RARγ is detectable from the onset of gastrulation (stage 10) while the first signal for RARβ is detected at the early tailbud stage (stage 25). At mid-gastrula stage (stage 11), RARα is expressed as a narrow ring around the blastopore. As gastrulation proceeds, expression intensifies and the signal around the blastopore widens preferentially on the dorsal side except in the midline, which exhibits a low level of transcripts. During neurulation (from stage 14), transcripts are found predominantly in the neurectoderm, evenly distributed along the anterior-posterior axis, with the exception of a region at the anterior end for which transcripts are largely reduced. At the tailbud stage (stage 30), RARα is predominantly expressed in the spinal cord and the posterior hindbrain, in the eye and the posterior branchial arches. During gastrulation (stage 10), expression of RARγ is more widespread than RARα expression. Transcripts are present in the mesodermal marginal zone as well as in the ectoderm. By the neurula stage (from stage 14), the staining separates into anterior and posterior domains, thus creating a gap with no RARγ transcripts. Expression remains localized to the posterior and anterior ends of the embryo at tailbud stages (stage 30) and is mainly restricted to the branchial arches and the tip of the tailbud. RARβ transcripts are detected at much lower level than RARα and RARγ at the examined stages. The signal is restricted to the caudal part of the hindbrain and the anterior spinal cord. At the late tailbud stage (stage 32), RARβ is strongly expressed in the most posterior branchial arches. d, dorsal views; l, lateral views; f, frontal views.
(2.8 MB TIF)

## **Abbreviations:**

ATRA: all-*trans* retinoic acid
BMS[number]: Bristol-Myers Squibb synthesized retinoid [number]
E[number]: embryonic day [number]
H[number]: helix [number]
LBD: ligand-binding domain
LBP: ligand-binding pocket
RA: retinoic acid
RAR: RA receptor
